# Identification of a Prognostic ceRNA Network Regulating TMBIM6 in Prostate Adenocarcinoma via Integrated Bioinformatic Analysis

**DOI:** 10.3390/ijms27010213

**Published:** 2025-12-24

**Authors:** Mst Sahida Khatun, Mohammad Mamun Ur Rashid, Muhammad Kamal Hossain, Hyung-Ryong Kim

**Affiliations:** 1 Department of Pharmacology, College of Dentistry, Jeonbuk National University, Jeonju 54907, Republic of Koreakamalhossain@jbnu.ac.kr (M.K.H.); 2 School of Pharmacy and Institute of New Drug Development, Jeonbuk National University, Jeonju 54896, Republic of Korea; 3 Non-Clinical Evaluation Center Biomedical Research Institute, Jeonbuk National University Hospital, Jeonju 54907, Republic of Korea; 4 Department of Pharmacy, Faculty of Pharmaceutical Sciences, University of Science and Technology Chittagong, Chittagong 4202, Bangladesh

**Keywords:** TMBIM6, PRAD, ceRNA network, miRNA, TCGA, GEO

## Abstract

TMBIM6, a transmembrane BAX inhibitor motif containing six proteins, is correlated with tumor progression and metastasis. While its correlation in several malignancies has been shown, its expression in prostate adenocarcinoma (PRAD) is unclear. In this work, we explored, using integrated bioinformatics, a novel ceRNA network of TMBIM6 involved in PRAD prognosis. According to TCGA and GEO datasets, we proposed a new TMBIM6/hsa-miR-222-3p/DHRS4-AS1 ceRNA axis associated with PRAD prognosis. The network was estimated by differential expression, correlation, and survival analysis. Co-expression analysis was used to identify pathways involved in tumor progression, and immune infiltration analysis suggested that there is a correlation between the expression of TMBIM6 and the abundance of epithelial cells. Overall, these results indicate that the DHRS4-AS1/hsa-miR-222-3p/TMBIM6 axis is involved in the progression of PRAD and may act as a prognostic biomarker. Our results provide a foundation for further experimental validation and potential clinical translation.

## 1. Introduction

An estimated 299,010 new cases and 35,250 deaths from prostate adenocarcinoma (PRAD) are anticipated in 2024 alone, making it the most common cancer in males and the second largest cause of cancer-related mortality in the US [1]. Although the epidemiological landscape shows a constant prevalence over the previous 40 years, there is still a worrying trend of advanced-stage diagnoses, which makes treatment results more difficult to manage [2]. The limits of prostate-specific antigen as a diagnostic tool, which lacks specificity and sensitivity and may result in overdiagnosis and overtreatment, are one of the current issues in prostate cancer care [3]. Furthermore, because there are currently few viable treatment options, the onset of castration-resistant prostate cancer (CRPC) substantially impairs prognosis [2]. In order to improve diagnostic precision and therapeutic success in the treatment of prostate cancer, these issues require continued investigation into new biomarkers and therapeutic approaches [3].

The concept of the competing endogenous RNA (ceRNA) network was first proposed by Salmena et al. in 2011, hypothesizing that such different RNA species, including long non-coding RNA (lncRNAs), pseudogenes, and circular RNAs, modulate the expression of each other through a competing process for shared binding sites of micro RNAs (miRNAs), also named micro RNA response elements [4,5]. The theoretical framework of the ceRNA network suggests that once a miRNA is bound by one RNA molecule, it may inhibit a miRNA from binding with its target mRNA, thereby regulating the expression of such mRNA. Such a regulatory mechanism has been verified to play a significant role in many biological processes, including cancer development and spread of cancer [6,7,8]. In cancer biology, lncRNAs are becoming more widely acknowledged as important regulators, especially in prostate cancer. Through several processes, including chromatin remodeling and interactions with signaling pathways, they affect both gene expression and the behavior of tumors. According to research [9,10], lncRNAs could function as ceRNAs, sequestering microRNAs and preventing them from suppressing their target mRNAs. ceRNA networks have garnered considerable interest in prostate cancer because of their role in the dysregulation of important genes and pathways linked to carcinogenesis [11,12].

TMBIM6, also commonly known as BAX-inhibitor 1 (BI-1), has been related to the activation of mechanistic target of rapamycin complex 2 (mTORC2) and AKT, implicated in tumor development [13]. Identification of a prognostic indicator will contribute significantly to further care and outcomes for prostate cancer patients. Considering the potential of TMBIM6 as a prognostic marker in prostate cancer, a comprehensive investigation using bioinformatics analysis is essential. Bioinformatics had previously identified the key players and the molecular pathways that are responsible for the tumorigenicity of the human prostate cancer [14]. Moreover, with the help of these bioinformatics methods, the participation of many genes and pathways in the development and progression of prostate cancer has been unraveled [15]. Among them, TMBIM6 has drawn considerable attention for its involvement in the progression of a wide range of cancers, including prostate cancer [13,16,17]. Previous study demonstrated that the downregulation of TMBIM6 triggers programmed cell death in prostate cancer cells, and therefore this protein might be a promising target for therapy [18]. However, the role of TMBIM6 in the progression of prostate cancer cells has not been thoroughly explored. Therefore, this study aims to investigate the specific function of TMBIM6 in the progression of prostate cancer by identifying the ceRNA network, which has provided new therapeutic avenues for cancers.

Moreover, ceRNA networks have been shown to influence the anti-tumor immune landscape in prostate cancer by controlling immune responses within the tumor microenvironment. Immune infiltrates and immune evasion strategies in prostate cancer can be influenced by ceRNA networks through modifying the expression of immune-related genes, ultimately impacting patient outcomes and the course of the illness [19].

Hence, the present study seeks to elucidate the function of TMBIM6 in the progression of PRAD by integrated bioinformatics analysis. Particularly, we speculate that TMBIM6 is modulated by a ceRNA network composed of lncRNAs and miRNAs and participates in disease progression and prediction. As far as we know, we are the first to find and describe the DHRS4-AS1/hsa-miR-222-3p/TMBIM6 axis as a possible prediction biomarker for prostate adenocarcinoma. This newly identified regulatory axis may provide important insights into the underlying biology of prostate cancer and could ultimately inform the development of more refined diagnostic tools and therapeutic interventions.

## 2. Results

### 2.1. Study Flow of Constructing the TMBIM6-Related ceRNA Network in PRAD

**Figure 1:**
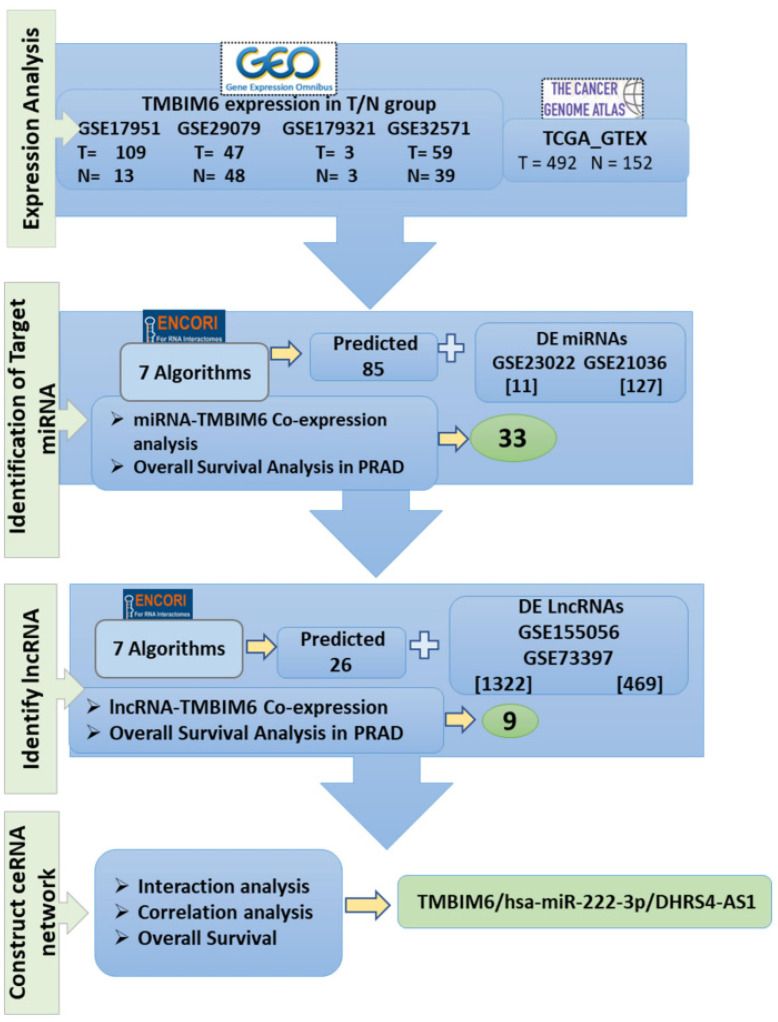
Flow diagram of construction and analysis of ceRNA network.

To evaluate the potential regulatory network of TMBIM6 in PRAD, we conducted a comprehensive analysis integrating data from multiple databases (Figure 1). Initially, differential expression analysis of TMBIM6 was performed using data from four GEO datasets (GSE17951, GSE20979, GSE179321, and GSE32571) and TCGA_GTEx, comparing tumor (T) and normal (N) tissue groups (Figure 1). Next, to identify miRNAs targeting TMBIM6, we utilized the ENCORI database, which applies seven different prediction algorithms, yielding 85 predicted miRNAs. These were intersected with differentially expressed (DE) miRNAs from GSE23022 and GSE20136, resulting in 33 candidate miRNAs. Co-expression analysis and overall survival analysis in PRAD were also performed (Figure 1). Subsequently, to identify upstream lncRNAs that may regulate these miRNAs, 26 candidate lncRNAs were predicted to use ENCORI and were cross-referenced with DE lncRNAs from GSE155056 and GSE73397, leading to the identification of 9 lncRNAs. These were further subjected to co-expression and survival analysis (Figure 1). Finally, interaction, correlation, and overall survival analyses were used to construct a competing ceRNA network. The resulting ceRNA axis, TMBIM6/has-miR222-3p/DHRS4-AS1, was identified as potential regulatory mechanism involved in prostate cancer progression (Figure 1).

### 2.2. TMBIM6 Is Overexpressed in Prostate Cancer Tissue

**Figure 2:**
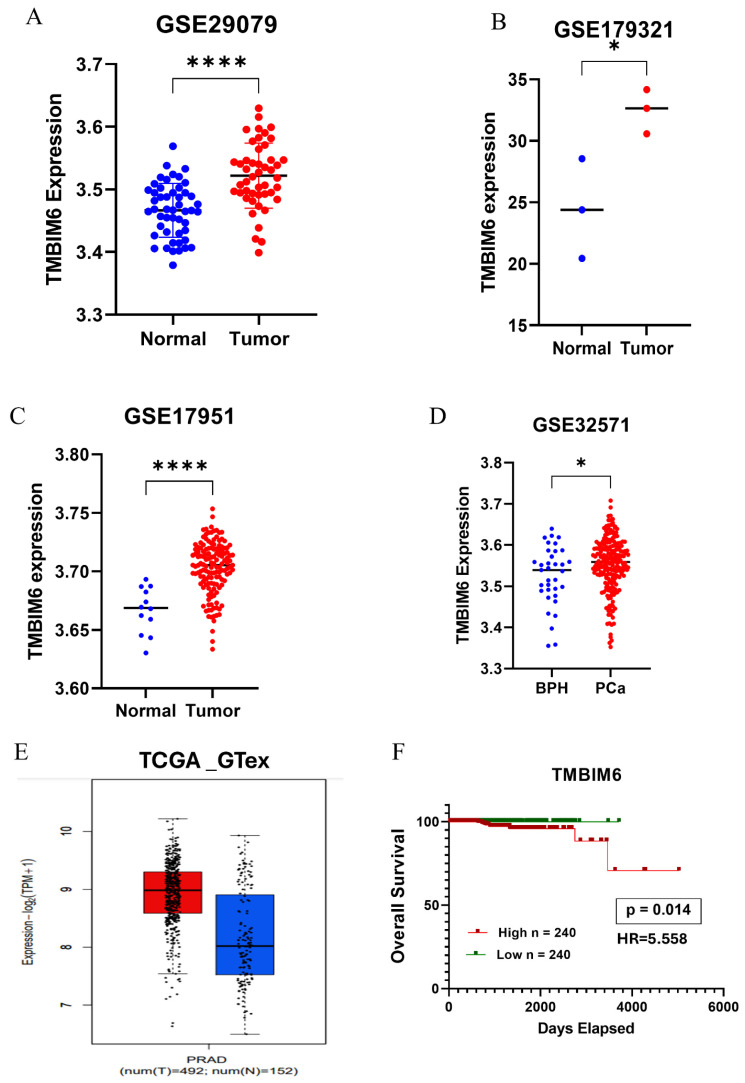
TMBIM6 is overexpressed in prostate cancer tissue. (**A**) Expression level of TMBIM6 in prostate cancer tissue compared to normal tissue by the GSE29079 dataset. (**B**) Expression level of TMBIM6 in prostate cancer tissue compared to normal tissue by the GSE179321 dataset. (**C**) Expression level of TMBIM6 in prostate cancer tissue compared to normal tissue by the GSE17951 dataset. (**D**) Expression level of TMBIM6 in prostate cancer tissue compared to normal tissue by the GSE32571 dataset. Expression levels are measured as log2 (fold change) from microarray experiments. (**E**) Expression level of TMBIM6 in prostate cancer tissue compared to normal tissue by the TCGA_GTEx dataset. (**F**) Overall survival plot of TMBIM6 in by TCGA-PRAD data. Statistical significance represented as * *p* < 0.05, and **** *p* < 0.0001, HR = Hazard ratio (Mantel-Haenszel).

We examined four GEO datasets (GSE29079, GSE179321, GSE17951, and GSE32571) to investigate the expression level of TMBIM6 in prostate cancer. The inclusion of both tumor and matched normal tissues, adequate sample size, consistent data quality, and relevance to prostate adenocarcinoma were the criteria used to select these datasets. TMBIM6 was discovered to be substantially upregulated in cancerous tissues as opposed to normal tissues in all four datasets (Figure 2A–D). Analysis of the TCGA-GTEx dataset also revealed increased expression of TMBIM6 in prostate cancer tissues, which is in line with these findings (Figure 2E). Additionally, Kaplan–Meier survival analysis of TCGA-PRAD data revealed that patients with high TMBIM6 expression exhibited significantly poorer overall survival compared to those with low expression (Figure 2F).

### 2.3. Identification of Target miRNA

**Figure 3:**
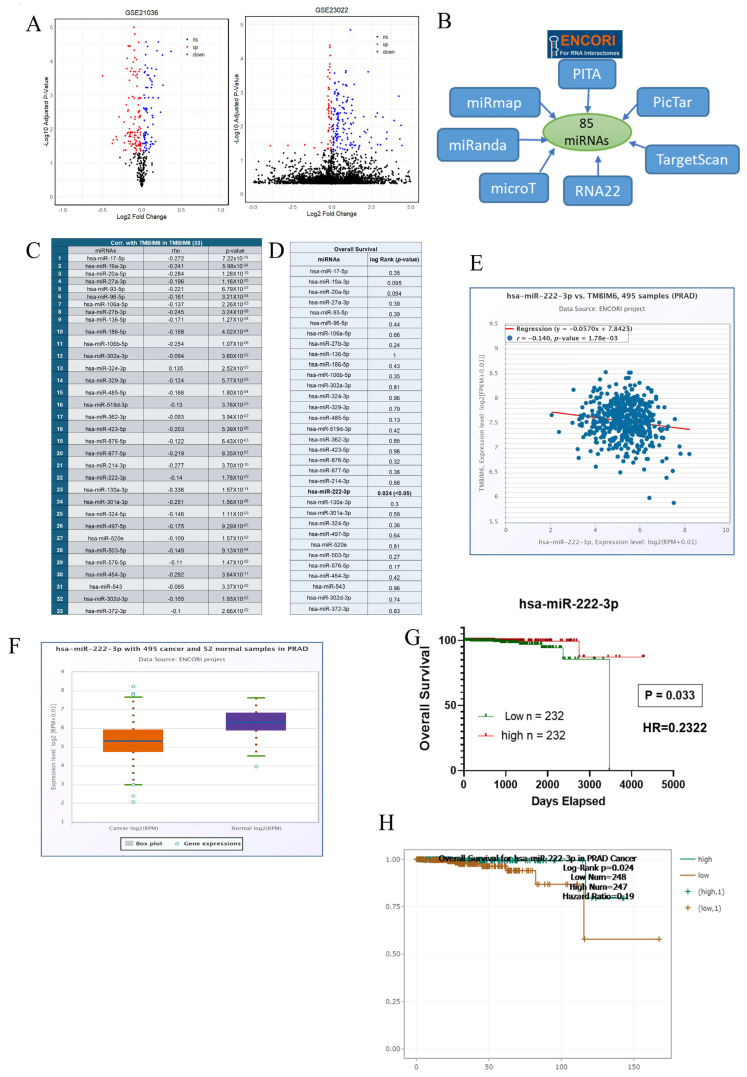
Identification of miRNA. (**A**) Volcano plot showing differentially expressed miRNAs in GSE21036 (92 up and 127 Down) and GSE23022 (153 up and 86 down) dataset. (**B**) A total of 85 miRNAs predicted by seven different programs. (**C**) A total of 33 miRNAs negatively correlated with TMBIM6 in prostate adenocarcinoma. (**D**) Overall survival analysis of 33 miRNAs in prostate adenocarcinoma. (**E**) Correlation plot between TMBIM6 and hsa-miR-222-3p in PRAD. (**F**) Expression plot of has-miR-222-3p in PRAD and normal tissue. (**G**) Overall survival plot of has-miR-222-3p by TCGA-PRAD data. (**H**) Overall survival plot of has-miR-222-3p in PRAD by ENCORI database.

In cancer study, it is important to identify the exact miRNAs targeting crucial genes in tumor biology and potential therapeutic targets. Herein, a series of analyses are performed in detail to uncover the miRNAs responsible for regulating TMBIM6. In this study, we predicted 11 and 127 miRNAs differentially expressed from two GEO datasets GSE23022 and GSE21036 (Figure 3A). Totally, 85 miRNAs were predicted by seven different programs (Figure 3B). All taken together DE and predicted miRNAs were used for further subsequent analysis. We conducted correlation analysis with TMBIM6 and estimated that 33 miRNAs were significantly negatively correlated (Figure 3C). Among these 33 miRNAs, we have inferred miRNA hsa-miR-222-3p, which has poor survival in the low expression group in PRAD, and the rest of the miRNAs were non-significant (Figure 3D). Figure 3E showed that has-miR-222-3p negatively correlated with TMBIM6 in PRAD (Figure 3E). We have also researched the expression level of has-miR-222-3p in PRAD and found that in the normal group has-miR-222-3p was significantly highly expressed (Figure 3F). Overall survival analysis showed the high expression group of hsa-miR-222-3p has better survival in PRAD (Figure 3G,H).

### 2.4. Identification of Target LncRNA

**Figure 4:**
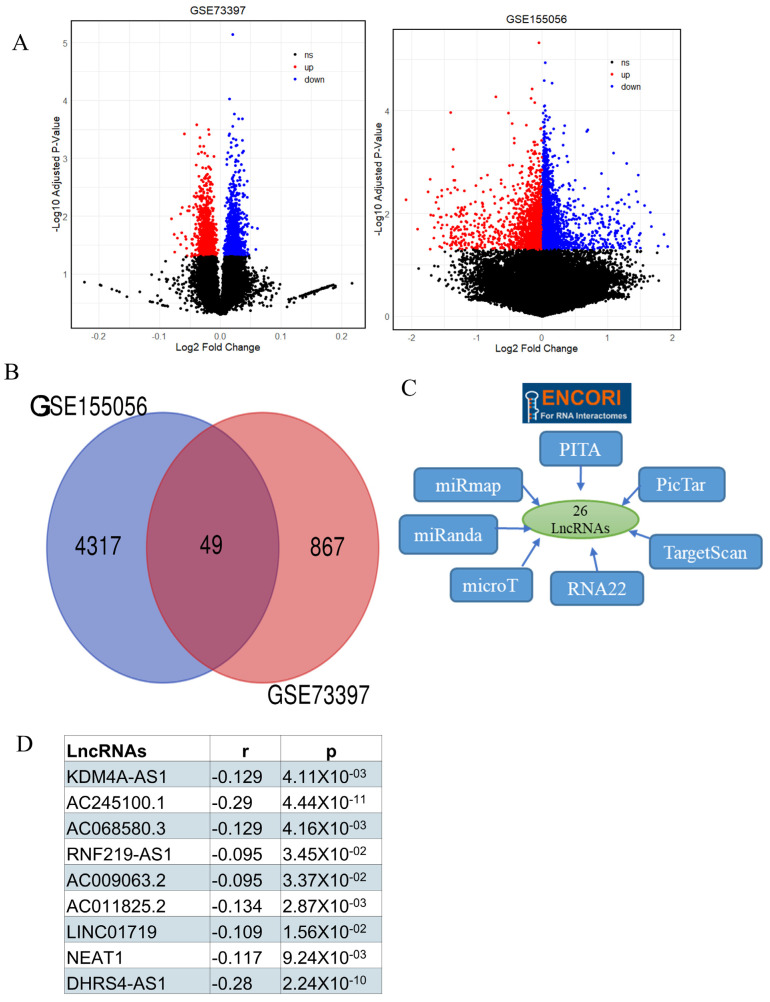
Identification of LncRNAs. (**A**) Volcano plot showing differentially expressed lncRNAs in GSE (92 genes are upregulated and 127 genes are downregulated) and GSE23022 (153 genes are upregulated and 86 genes are downregulated) dataset. (**B**) A total of 26 lncRNA were predicted by seven different algorithms by ENCORI. (**C**) Venn diagram showing 49 lncRNA commonly differentially expressed in PRAD. (**D**) Nine lncRNA were estimated, which are significantly negatively correlated with has-miR-222-3p in PRAD.

It is indispensable in cancer research to identify the lncRNAs that regulate key genes and map their roles in the ceRNA network for the uncovering of novel molecular mechanisms and potential therapeutic targets. Accordingly, we performed several analyses to determine the lncRNAs associated with TMBIM6 in their role within the ceRNA network in prostate cancer. First, we identified 1,322 and 469 differentially expressed lncRNAs from the GSE155056 and GSE73397 datasets, respectively (Figure 4A). Among these, 49 lncRNAs were commonly differentially expressed in both datasets (Figure 4B). We have used several algorithms to predict target lncRNAs and 25 lncRNAs were predicted by seven algorithms (Figure 4C). After that we performed correlation analysis and estimated that nine lncRNAs were significantly negatively correlated with has-miR-222-3p (Figure 4D).

### 2.5. Construct ceRNA Network

**Figure 5:**
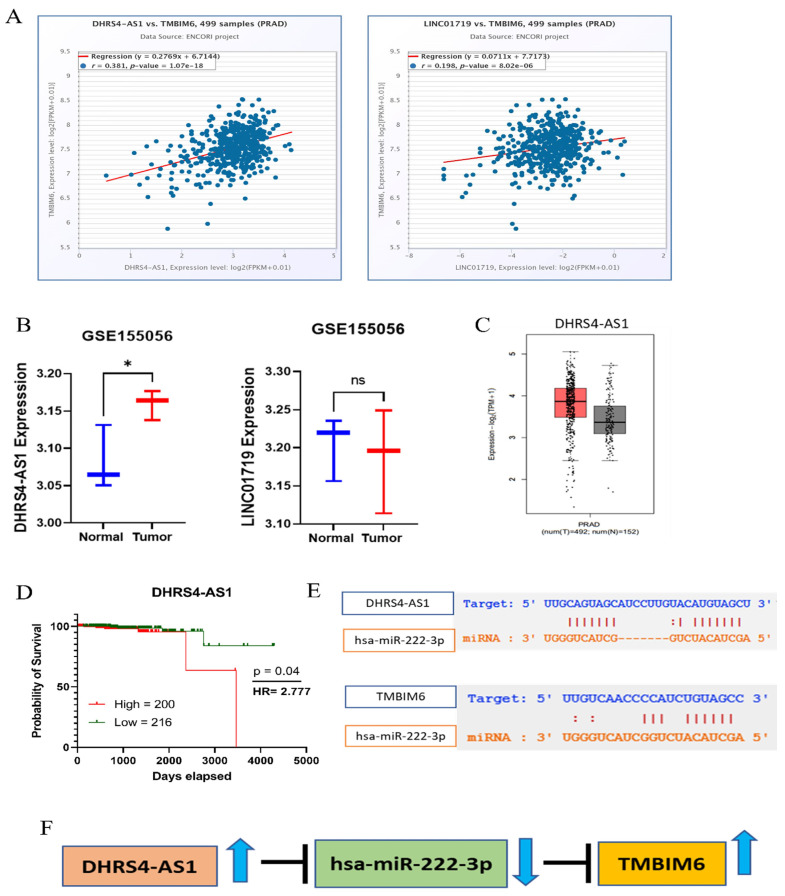
Construct ceRNA network. (**A**) Two positively correlated lncRNAs, i.e., DHRS4-AS1 and LINC01719 with TMBIM6 in PRAD. (**B**) Boxplot showing the differential expression level in PRAD and normal tissue of DHRS4-AS1 and LINC01719 by GSE155056 dataset of PRAD. (**C**) Boxplot showing differential expression level of DHRS4-AS1 in PRAD compared to normal checked by GEPIA webtool based on TCGA_GTex data. (**D**) Overall survival plot of DHRS4-AS1 in PRAD (**E**) Figure showing the interaction of hsa-miR222-3p with DHRS4-AS1 and TMBIM6. (**F**) Figure of construction of ceRNA network. Statistical significance represented as * *p* < 0.05, ns as not significant, HR = Hazard ratio (Mantel–Haenszel).

To construct the ceRNA network further, we performed several analyses. Among the nine lncRNAs we predicted two lncRNAs, which were significantly positively correlated with TMBIM6 in PRAD (Figure 5A). Furthermore, we have analyzed the expression level of these two lncRNA in the GSE155056 dataset, and DHRS4-AS1 significantly highly expressed (Figure 5B). However, LINC01719 does not express in the PRAD dataset (Figure 5B). Eventually, we have proposed DHRS4-AS1 as lncRNA for constructing ceRNA network of TMBIM6. Moreover, we have also checked the expression level of DHRS4-AS1 by GEPIA tool and obtained the same result (Figure 5C) and performed overall survival analysis of DHRS4-AS1 in PRAD, and results showed that high expression groups have poor survival (Figure 5D). We have checked the interaction between lncRNA-miRNA and miRNA-mRNA to see the interaction region (Figure 5E). Finally, we have constructed ceRNA network of PRAD (Figure 5F).

### 2.6. Immune Infiltration Analysis

**Figure 6:**
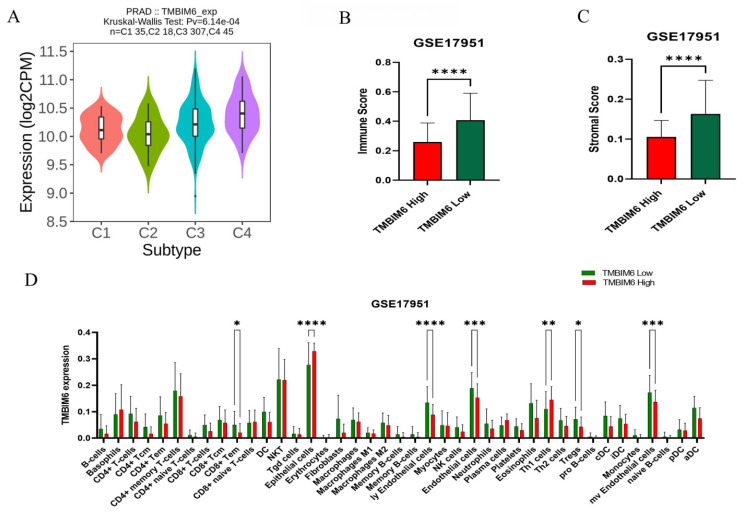
Immune infiltration analysis. (**A**) Association between TMBIM6 expression and immune subtypes of prostate adenocarcinoma. (**B**) Relationship between TMBIM6 and immune score. (**C**) Relationship between TMBIM6 and stromal score. (**D**) Correlation between TMBIM6 high and low expression group and 34 types of immune and stromal cells * *p* < 0.05, ** *p* < 0.005, *** *p* < 0.0005, and **** *p* < 0.0001.

The correlation between immune infiltration and TMBIM6 in prostate cancer is revealed by analyzing immune cell infiltration in the tumor microenvironment (TME). We investigated the function of TMBIM6 gene expression in PRAD immune and molecular subtypes. The findings showed that in PRAD, TMBIM6 was highly expressed in C1, C3, and C4 subtypes (Figure 6A). Furthermore, 34 different types of immune and stromal cells were analyzed PRAD using the xCELL algorithm, a ssGSEA-based algorithm. This analysis revealed that TMBIM6 high expression is associated with epithelial cells and Th1 cells. (Figure 6B). We investigated the possible connection between immune cell infiltration and TMBIM6 expression in prostate adenocarcinoma. The findings indicated that the group with low-expressing PRAD had higher ImmuneScore and StromalScore (Figure 6C,D).

### 2.7. Identification of Co-Expressed Genes of TMBIM6 in Prostate Cancer

**Figure 7:**
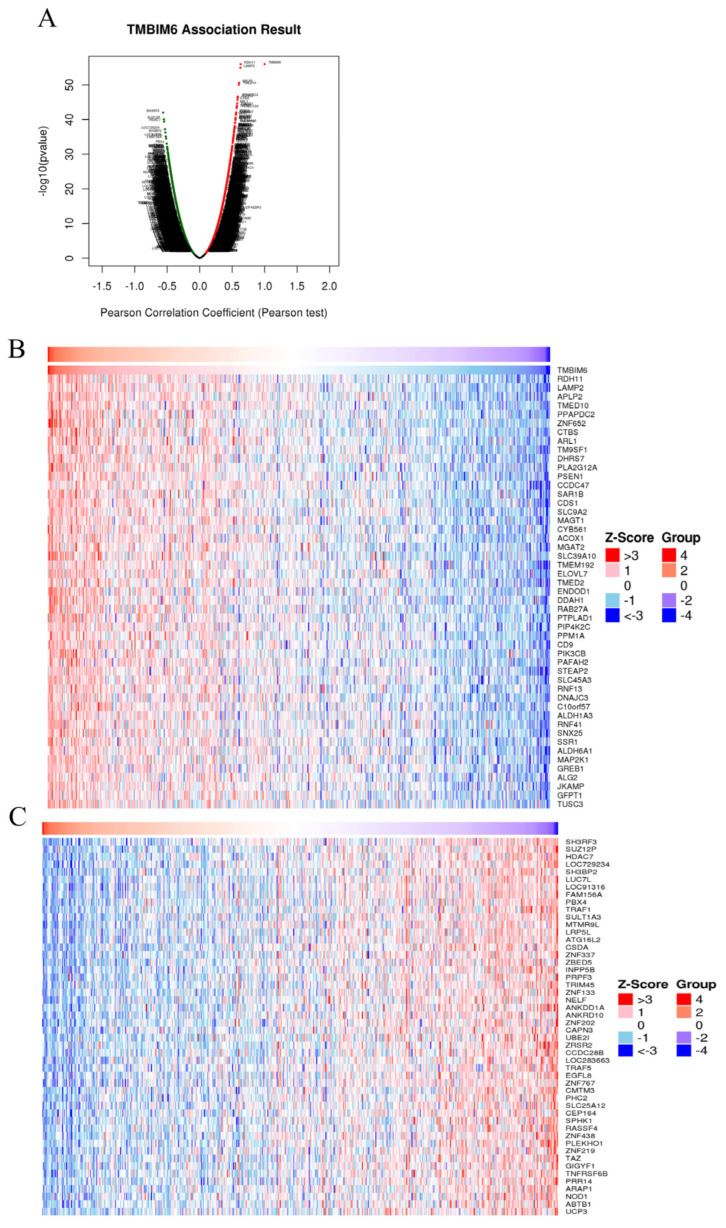
Identification of co-expressed genes of TMBIM6 in prostate adenocarcinoma (**A**) Volcano plot shows co-expressed genes of TMBIM6 in prostate adenocarcinoma. (**B**) Heatmap represents the top 50 positively co-expressed genes with TMBIM6 in PRAD. (**C**) Heatmap displays top 50 negatively co-expressed genes with TMBIM6 in PRAD.

Co-expression analysis identified a significant number of genes that are co-expressed with TMBIM6 in prostate cancer. More precisely, 4649 genes showed significant positive co-expression, and 8229 genes were negatively co-expressed with TMBIM6 (Figure 7A). The top 50 genes with positive co-expression are displayed in a heatmap in (Figure 7B), while the top 50 genes with negative co-expression are shown in (Figure 7C).

## 3. Discussion

In this work, we systematically assembled and explored a ceRNA network that governs TMBIM6 in PRAD. The TMBIM6/has-miR-222-3p/DHRS4-AS1 regulatory axis was determined as a probable prognostic module. Expression and correlation studies corroborated that TMBIM6 is negatively correlated against miR-222-3p, while DHRS4-AS1 was positive against TMBIM6 and negative against miR-222-3p. The survival analysis also revealed that higher expression of miR-222-3p was associated with better prognosis. Co-expression and immune infiltration studies revealed further correlations with STEAP2, PIK3B, and epithelial cells, suggestive of TMBIM6 impacting cancer progression in multiple manners.

Several ceRNA regulatory networks have been reported on PRAD. For example, Guo, L. et al., demonstrated that lncRNA SNHG3 can sponge miR-222-3p to regulate TK1 expression, suggesting a functional ncRNA network influencing prostate cancer progression [20]. Similarly, Autophagy-related lncRNAs were shown to regulate competing endogenous RNA networks and predict survival outcomes in prostate cancer, highlighting their functional and prognostic significance [21]. A recent study by Taheri et al. reported four ceRNA regulatory axes—ADAMTS9-AS2/miR-150/PRKCA, ADAMTS9-AS2/miR-150/MMP14, MEG3/miR-150/PRKCA, and MEG3/miR-150/MMP14—highlighting miR-150 as a central regulatory molecule strongly associated with patient survival [22]. Our findings extend this body of work by focusing on TMBIM6, a gene previously less studied in PRAD but known to regulate cell death and stress responses in other cancers. To our knowledge, this is the first report proposing a TMBIM6-centered ceRNA axis in prostate cancer.

miR-222-3p is involved in various cancers, wherein it usually facilitates tumorigenesis. MiR-222-3p was reported to be involved in tumor progression in various cancer types. In breast and thyroid cancer, overexpressed miR-222-3p induces proliferation, migration, and invasion, while in castration-resistant prostate cancer, exosomal miR-222-3p upregulates such malignant behaviors by activating mTOR signaling [23,24,25]. However, in this current work in primary PRAD, downregulation of miR-222-3p in tumors was detected, and higher expression correlated with favorable prognosis, imparting a probable tumor-suppression upon it in this scenario [20]. In the TMBIM6/has-miR-222-3p/DHRS4-AS1 axis, DHRS4-AS1 might function as a ceRNA sequestering miR-222-3p, hence regulating TMBIM6 expression. Our results show that this regulatory axis could render protection in PRAD, in contrast with oncogenic roles of miR-222-3p in other malignancies and CRPC. This lends emphasis to the context-dependent role of miR-222-3p and places importance upon cancer-specific studies of ceRNA networks while considering potential prognostic markers.

The lncRNA DHRS4-AS1 has been shown to act differently in cancers, both as a tumor suppressor and oncogene depending on the scenario. For instance, DHRS4-AS1 inhibits proliferation and stemness in non-small-cell lung cancer by sequestering miR-224-3p and upregulating TP53 and TET1 and induces apoptosis in hepatocellular carcinoma by the miR-522-3p/SOCS5 pathway [26,27]. Likewise, in thyroid and gastric cancers, DHRS4-AS1 can suppress cellular proliferation and induce apoptosis by miRNA-based ceRNA activity [28,29]. In contrast, DHRS4-AS1 downregulation in clear-cell renal cell carcinoma is associated with adverse prognosis, revealing its tumor-suppressive activity [30]. Our work illustrated that DHRS4-AS1 expression is elevated in PRAD tissues versus normal controls, correlated with TMBIM6 in a positive manner, and inversely with miR-222-3p. The model asserts support of oncogenic activity in prostate cancer, in contrast to that in other cancer types, and underlines novelty in our ceRNA axis.

Of genes that are co-expressed with TMBIM6, of particular interest were STEAP2 and PIK3B. STEAP2, a six-transmembrane epithelial antigen of the prostate, is uniformly overexpressed in PRAD and associated with higher Gleason grades and aggressive phenotypes, which is in line with its strong positive correlation with TMBIM6 in this study [31,32,33]. PIK3B, as a key member of the PI3K/AKT signaling cascade, is typically dysregulated in prostate cancer and fosters excess tumor viability and therapy refractoriness [33,34]. Co-expression of TMBIM6 alongside PIK3B suggests that TMBIM6 may contribute to PI3K/AKT signaling activation, in turn facilitating further malignant progression of the tumors. Interestingly, in addition to its prognostic significance, our correlation analysis provides further insight into the biological role of TMBIM6 in prostate cancer. The genes most positively associated with TMBIM6 expression, including LAMP2, APLP2, TMED10, PPAPDC2, ZNF652, and CTSB, are primarily involved in lysosomal and ER trafficking, membrane dynamics, and metabolic resilience—pathways that facilitate tumor survival under cellular stress conditions [35]. Such enrichment suggests that TMBIM6 upregulation may reinforce proteostasis and metabolic adaptation programs that are advantageous for tumor progression. Conversely, several negatively correlated genes, such as SUZ12P, HDAC7, LUC7L, and TRAF1, participate in chromatin regulation, RNA maturation, and immune-related signaling [36]. Their reduced expression in TMBIM6-high tumors may indicate attenuation of immune activation and stromal responsiveness. Together, these opposing transcriptional patterns are consistent with the emerging notion that elevated TMBIM6 expression contributes to an immunosuppressive tumor microenvironment in PRAD, promoting tumor cell fitness while dampening anti-tumor immune pressure.

Immune infiltration analysis suggested that up-regulated TMBIM6 expression is associated with higher epithelial cell enrichment in the tumor microenvironment. Tumors with low TMBIM6 expression exhibit significantly higher immune and stromal scores, indicating increased infiltration of non-tumor cells within the tumor microenvironment (Figure 6B,C). Consistent with this, Figure 6D reveals that multiple immune and stromal cell populations are enriched in the TMBIM6-low group. The results agree with known roles of epithelial–mesenchymal transition (EMT) in prostate cancer progression, marked by loss of inter-cell adhesions in epithelial cells in conjunction with acquisition of greater migratory and invasive properties [37,38]. Previous studies have shown that EMT is associated with higher Gleason grades, higher metastatic potential, and therapy refractoriness in PRAD such that stromal–epithelial crosstalk additionally enhances tumor aggressivity [39,40]. Through demonstration of a link of TMBIM6 expression with epithelial infiltrations, this work suggests TMBIM6 fosters EMT processes, thereby underpinning enhanced invasiveness and higher risks of metastatic spread in prostate adenocarcinoma.

Together, these findings imply that this ceRNA axis TMBIM6/miR-222-3p/DHRS4-AS1 might serve as a new prognostic biomarker in prostate adenocarcinoma. Unlike previous works, which often concentrate heavily on ceRNA networks in general, this work, by contrast, places an emphasis upon a single, so far uncharacterized, regulatory module centered upon TMBIM6. At a more clinical and translational level, defining this regulatory pathway may enable the development of robust prognostic biomarkers and, importantly, guide the discovery of novel therapeutic targets that could ultimately refine and personalize prostate cancer treatment.

## 4. Materials and Methods

### 4.1. Data Preparation and Processing

Gene expression omnibus (GEO) is a global public data repository for functional genomic data from next-generation sequencing (NGS) and microarrays [41]. We have retrieved expression datafile and sample information datafile by R software (version 4.3.1; R Foundation for Statistical Computing, Vienna, Austria) by using GEOquery package from 4 GEO datasets, GSE29079, GSE179321, GSE17951, GSE32571 [42,43,44,45,46,47]. Data was preprocessed by dplyr package. The cancer genome atlas (TCGA) is an open-access database that contains information on 39 different tumor types from over 11,000 samples [48]. TCGA-PRAD dataset was downloaded and preprocessed by TCGABiolinks, Summarized Experiment packages.

Processed expression matrices derived from GEO (series_matrix files) and TCGA (FPKM values) were utilized for subsequent analyses. Each dataset underwent normalization employing platform-specific methodologies (GEO: RMA/quantile normalization; TCGA: TPM values transformed using log2(TPM + 1)). For the purpose of cross-platform comparison, expression values were converted into z-scores on a gene-by-gene basis (subtracting the mean and dividing by the standard deviation), thereby centering and scaling each gene’s distribution across samples. Differential expression analyses were conducted for each dataset, applying thresholds of |log2FC| > 1 and false discovery rate (FDR) < 0.05. To assess reproducibility among datasets, we executed (i) correlation analyses of log2 fold-changes across datasets and (ii) direct comparisons of the expression levels of key genes, utilizing boxplots. These methodologies revealed consistent expression patterns for TMBIM6 and the DHRS4-AS1/hsa-miR-222-3p axis across independent cohorts.

### 4.2. Expression Analysis

We analyzed TMBIM6 expression across various datasets to explore its potential role in prostate cancer. Expression mining for TMBIM6 in four datasets from GEO was performed: GSE29079, which includes 47 cases of prostate tumor samples and 48 normal tissues; GSE179321, with three pairs of tumor and adjacent normal tissues [42,43,44]. Further, expression data are available from GSE17951 for 109 prostate tumor samples and 13 normal tissues and GSE32571 dataset consists of 59 and 39 tumors and normal samples were analyzed [45,46,47]. Web tool GEPIA2 (Gene Expression Profiling Interactive Analysis) has been used in TMBIM6 expression analysis for TCGA-GTex data analysis [49,50].

### 4.3. Co-Expression Analysis

Co-expression analysis was performed to investigate the possible gene networks of TMBIM6 by using the LinkedOmics database [51]. This database contains multiomics data from 11158 TCGA program participants, representing 32 different cancer types [51]. LinkFinder module, we used to execute Spearman’s correlation analysis and identified genes that were positively or negatively co-expressed with TMBIM6. The top 50 co-expressed genes were then represented in a heatmap.

### 4.4. Identification of lncRNAs and miRNAs

We conducted an in-depth analysis to investigate potential interactions between miRNAs and TMBIM6. First, using the ENCORI/starBase platform, we used seven different algorithms (PITA, RNA22, miRmap, microT, miRanda, PicTar, and TargetScan) to predict the potential miRNA targets of TMBIM6 [52,53]. Next, we used two GEO datasets, GSE23022 and GSE21036, to identify miRNAs that were differentially expressed in prostate cancer [54,55]. Then, the expression correlation between predicted microRNAs and differentially expressed microRNAs with TMBIM6 was analyzed in prostate cancer tissues, which identified 33 microRNAs that were negatively correlated with TMBIM6.

Accordingly, we have carried out a series of comprehensive analyses to explore the possibility of lncRNAs for constructing ceRNA network. Like our strategy in studying miRNA, we listed some predicted and differentially expressed lncRNAs in prostate cancer and investigated their correlation to target miRNA using the Starbase database. Nine lncRNAs were significantly negatively correlated with miRNA.

### 4.5. ceRNA Network Construction

To construct ceRNA network analyzed the expression of these nine lncRNAs in prostate cancer tumor tissues compared to normal tissues by GEPIA2, an online application that uses the GTEx projects [49,50]. We also checked the expression level of lncRNAs from GSE155056 dataset, preprocessed by R software and visualize by GraphPad Prism Version 10.2.3 [56]. Overall survival analysis is also performed by TCGA-PRAD data [57].

### 4.6. Immune Infiltration Analysis

The molecular subtypes of TCGA-PRAD samples (C1–C4) were obtained from Guo et al., 2022 [58], who classified PRAD into four immune-associated subtypes based on consensus clustering of transcriptomic profiles. TMBIM6 expression (log2CPM) was compared across subtypes using the Kruskal–Wallis test. TMBIM6 expression was analyzed deeply in immune infiltration to explore its relationship with immune cell infiltration in prostate cancer. With the use of data from TCGA, the xCELL algorithm, which is a single-sample Gene Set Enrichment Analysis (ssGSEA)-based method used to profile 64 distinct types of immune and stromal cells, was used to analyze [59,60]. We used the ESTIMATE algorithm [61] implemented in R to derive immune and stromal scores from TCGA-PRAD transcriptomic data. We focused on 34 types of immune and stromal cells to analyze their association with TMBIM6 expression in prostate cancer in this study.

### 4.7. Statistical Analysis

R software and GraphPad Prism were used for all statistical analyses. In particular, the log-rank test was utilized to generate Kaplan–Meier curves during survival analysis, and the Pearson correlation was employed during co-expression analysis. Two groups and several groups were compared using the unpaired *t*-test and two-way ANOVA, respectively. A *p*-value of less than 0.05 was deemed statistically significant.

## 5. Limitations

This study has several important limitations that should be carefully considered when interpreting the results. First, all analyses rely entirely on publicly available transcriptomic datasets, which differ in sample size, sequencing platforms, normalization pipelines, and clinical characteristics. Despite applying robust harmonization steps, residual batch effects and technical variability may influence expression patterns. Second, the ceRNA interactions proposed in this study are inferred from prediction algorithms and correlation analyses; such computational approaches cannot establish physical binding, competitive miRNA sequestration, or functional regulation. Third, immune and stromal observations derived from ESTIMATE and xCell represent relative enrichment rather than absolute cell counts or spatial architecture, meaning that tumor purity differences may underlie many observed patterns. Fourth, survival associations for TMBIM6, hsa-miR-222-3p, and DHRS4-AS1 were modest and may vary depending on dataset composition or cutoff strategy. Finally, the study lacks in vitro or in vivo validation, and therefore all conclusions should be interpreted as hypothesis-generating rather than mechanistic or definitive.

## 6. Future Directions

Based upon our findings and limitation of the study, research should experimentally validate the proposed DHRS4-AS1/hsa-miR-222-3p/TMBIM6 axis through in vitro binding assays, functional knockdown/overexpression studies, and in vivo prostate cancer models to confirm its mechanistic role. Integrating single-cell and spatial transcriptomic approaches will help resolve tumor purity effects and clarify cell-type–specific regulation of this ceRNA network. Larger, clinically annotated cohorts—ideally including proteomic and longitudinal data—will be essential to strengthen the prognostic value of TMBIM6 and its associated ceRNA components. Mechanistic investigations dissecting how this ceRNA network shapes oncogenic signaling, modulates therapeutic responsiveness, and drives tumor microenvironment remodeling could open new avenues for precision prognostics and targeted intervention in prostate cancer.

## Data Availability

No new datasets were generated or analyzed.
